# Equivalence Assessment Method of Forest Tourism Safety Based on Internet of Things Application

**DOI:** 10.1155/2022/1578005

**Published:** 2022-04-28

**Authors:** Yuan Qi, Xuan Gao

**Affiliations:** ^1^Department of Tourism, School of Tourism Management, University of Sanya, Sanya 572022, China; ^2^Department of Hotel Management, School of International Hospitality Management, University of Sanya, Sanya 572022, China

## Abstract

With the continuous development of the society, China's economy and technology have been greatly improved, and network technology has also been widely used in life. In China's forestry management, the use of Internet of Things technology has gradually formed a model, which has greatly helped the economic benefits of forests. In addition, with the rapid development of the tourism industry, the number of tourists has increased sharply, the tourism infrastructure and tourism management are relatively lagging behind, and tourism safety accidents have occurred from time to time. However, the application of IoT technology in forestry is still in its infancy, with a small scope of application and low technical level. Aiming at the uniqueness of forest management, this paper proposes the development direction and application planning of IoT in forest resource supervision and service, forest fire prevention and control and service, ecological environment monitoring, and forest tourism supervision and service. In addition, this paper also discusses the acquisition technology of geological disasters, air quality, meteorological conditions, passenger flow conditions, and traffic flow mainly involved in tourism safety from the macroperspective of the Internet of Things. At the same time, the specific application of these technologies in tourist attractions is discussed to provide some technical reference for the realization of scientific and safe tourism management.

## 1. Introduction

The Internet of Things is to use radio frequency identification, infrared sensors, global positioning systems, laser scanners, and other information sensing equipment to connect any item with the Internet according to the agreed protocol to exchange and communicate information to realize the intelligentization of items. A network is identified, located, tracked, monitored, and managed. The Internet of Things is an important part of the new generation of information technology [1–5].

Strengthening the research and development and industrial application of Internet of Things technology is conducive to accelerating the transformation of economic development mode, promoting industrial structure adjustment and optimization and upgrading, and has important strategic significance for seizing the commanding heights of future economic technology and enhancing industrial competitiveness. The state conducts early application demonstrations in six major fields, including urban intelligent transportation, public safety, environmental protection, agriculture, forestry, and smart home. It can be seen that the state has taken forestry informatization as the pillar and goal of modern forestry construction [6–9].

**Figure 1:**
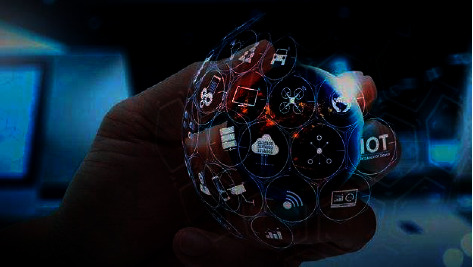
IoT.

The Internet of Things has been widely used in forest management, such as in forest fire monitoring systems, as shown in Figure 1. The Internet of Things technology provides three levels of services for forest fires to realize information conversion, mutual communication, and mutual operation. (1) Perception layer: the perception layer is the basis for ensuring the development and practical application of the Internet of Things. The bottom layer of information technology is to combine a series of forest fire-related information parameters such as RFID technology, infinite sensing technology, and remote sensing monitoring technology to transmit network information to the next layer. (2) Network layer: the network layer uses the existing communication links such as the Internet, ground GPRS, and satellite communication networks to receive, analyze, and process the data of the perception layer and store them in the data server. (3) Application layer: the application layer uses the analyzed and processed perception information to provide users with the customized information they need on the one hand and feedback to the bottom layer of the Internet of Things to achieve corresponding control [10–17]. The Internet of Things can do many things that humans cannot. This leads to greater efficiency, convenience, and in some cases even security. Devices process data faster than us.

In addition, IoT can also be used in the field of forest fire protection. The forest fire prevention business covers forest fire monitoring, forecasting, forecasting, emergency response, command, and other links. It is a complex emergency response system. The level of informatization in this field is relatively high. At present, there are more than 1,500 forest fire video monitoring points, more than 670 forest fire risk monitoring stations, more than 110 combustible material information collection stations, and more than 17,000 artificial observation platforms for fire-fighting teams. The application of more than 10,000 IoT-related technologies in forest fire prevention mainly includes three aspects: one is positioning, the other is video monitoring, and the third is abnormal temperature sensing [18–22].

The fire prevention system plays a very important role in forest management and can more effectively prevent the occurrence of fire. In the actual application process, it is necessary to deal with the relationship between each network layer, clarify the division of labor at each level, and comprehensively grasp the relevant factor. The management of data should be able to meet the requirements of the network information mechanism, establish a unified command center, and systematically classify the actual situation of the fire. If necessary, a corresponding database can be set up to lay a certain foundation for the development of the forest system. In addition, it is to realize the sharing of resources, which is also the key point of the application of the Internet of Things. The establishment of the forest fire prevention system will also provide certain help for the sharing. In the process of data sharing, if there is a sudden situation, the first thing you should do is to locate it. When the command center receives the corresponding information, it will determine the most appropriate method and reasonably allocate personnel for rescue. The spread of fire to the greatest extent is prevented, and the damage to the forest is reduced [23–28].

**Figure 2:**
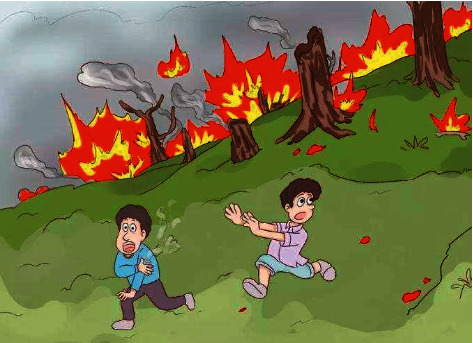
Forest fire management and monitoring.

In the process of modern forest fire management and monitoring (as shown in Figure 2), the application of monitoring software is a very important content, which mainly includes several parts. The first is the data management module. Combined with the actual format, the collected data and information are completely saved, and the relevant data query methods are set at the same time. In addition, it can monitor the historical weather and fire conditions in the region, and can also provide the risk level that may exist in the forest in the near future, and take timely response measures. First, an accurate statistical table can be built to provide a certain basis for later work; second, the data query module, this part can also save the information collected by the sensor and can also provide the function of querying the map; third, the monitoring module, through the transmission network of application data, the corresponding information can be received and then displayed accurately, and the application network of the sensor can be controlled to a certain extent; fourth, the module of data analysis, by analyzing the on-site information collected by the sensor, combined with the actual situation, a scientific and reasonable prediction is synthesized to provide certain auxiliary functions.

However, IoT technology has problems and gaps in forest management, such as resource management. Because the Internet of Things technology is an emerging industry with relatively few applications in practice, it is currently in the stage of exploratory development. The main problems that arise are (1) the business level is low, the data resources are few, and the amount that can be shared cannot be satisfied. Practical application is not conducive to finding and solving problems; (2) the management method is not advanced enough, and the way of use is relatively simple; (3) the database and the application system cannot reach the same standard and cannot form a unified model. It can save the time. Because it reduces manpower, it definitely saves time. Time is the main factor that can be saved through an IoT platform.

In the field of forest fire prevention, we have the following: (1) The general forests affected by the signal are located in relatively backward and remote areas, and there are almost no settlements in the forest area covered by alpine forests. The network and communication are all far away words: lack of professional forest network in fire. It is difficult to transmit information when it occurs. (2) Monitoring system problems: our country lacks a systematic solution in forest fire monitoring and has not introduced relevant equipment to forest fire monitoring. The method used is still the traditional manual wait-and-see approach, and the accuracy and timeliness cannot be compared with advanced technical equipment. (3) Video sharing problem: at present, the provincial forest fire video monitoring system has not been connected with the State Forestry Administration, which affects data sharing, emergency response, and unified command and dispatch. (4) Communication and monitoring equipment problems: at present, the information equipment level of the fire-fighting team is very low. The mobile terminal function of the fire-fighting team is relatively simple and lacks functions such as positioning and communication. (5) Data intelligent analysis problems: in the face of a large amount of information obtained from forest fire monitoring, the current intelligent analysis capability in the background is very low, which seriously affects the support and reference for decision-making. In the process of IoT application, its signal will be affected. Because many forests are located in relatively remote areas, the surrounding population is relatively small, and they are all mountainous areas. The network construction is also very backward, the communication is relatively inconvenient, and there is no professional internal network in the forest area. In the event of a fire situation, the speed of information transmission will be affected; second, in the fire monitoring system, the entire system is not perfect, there are still some loopholes in the middle, some advanced equipment has not been applied to the monitoring system, and more traditional methods are used. The accuracy and real-time performance are far from satisfying the status quo, and the effect on forest fire prevention work is minimal; third, due to the incomplete construction of the whole, there is no good connection between the provincial-level video surveillance and the country, and the data sharing has not been established; emergency, command, and other work will have a great impact; finally, the problem of communication equipment is obtained. Because the forest is in a relatively remote area, the equipment for forest firefighting organizations is relatively backward, especially the level of informatization. Many team members have relatively single terminals and do not have the functions of positioning and real-time communication that modern equipment should have. It is because IoT can minimize human labor. That is, when IoT devices interact and communicate with each other and accomplish a large number of tasks, they can minimize human labor.

In the field of ecological environment monitoring and forest tourism supervision, (1) most of the existing digital acquisition equipment does not have wireless transmission function, the coverage is small, and the amount of information is limited; (2) forest area communication is a big constraint; (3) forest area is positioning. The problem is difficult to solve effectively. The proposed IoT has the following advantages: efficient resource utilization, knowing how each device functions, works, improves the efficient utilization of resources, and monitors natural resources.

## 2. Assessment Method

The fuzziness of things often refers to the uncertainty contained in objective things. In daily life and production, various ambiguity problems are often encountered, and ambiguity problems often have the characteristics of “one and the other” and have strong uncertainty. For example, the representation of weather is described by “sunny” and “yin”, the representation of a system state is described by “good” and “bad”, and it is described in a qualitative way of “either or the other”. The real state of uncertainty problems often has a large deviation or even distortion. At present, the risk decision-making of many complex systems involves a large number of uncertain problems. To realize the risk decision-making of uncertain complex systems, it is impossible to solve the uncertain problems qualitatively with fuzzy concepts.

Fuzzy set is a set of things with unclear boundaries and special properties. Fuzzy set is a simple mathematical tool that can transform the adjective of difference into a membership function and then use the method of logical operation to solve the problem from simple to rising to complex puzzles. In ordinary set theory, the relationship between an element *x* and a set *S* can only be *x* ∈ *S* or *x* ∉ *S*. In addition, the relationship between an element *x* and a set *S* can also be expressed by the following logical relationship:

(1) $$\begin{array}{c} S\left(x\right)=\left\{\begin{array}{c} 0x\notin S\\ 1x\in S \end{array}\right., \end{array}$$

where 0 and 1 are the eigenvalues of the logical relationship between the element *x* and the set *S*.

The fuzzy comprehensive assessment method considers that the security risk of forest tourism is a complex uncertainty system, which is mainly manifested in the inherent ambiguity of risk because risk is a relative concept. Fuzzy mathematics is to evolve the eigenvalue {0, 1} of the logical relationship between element *x* and set *S* into any real value between [0, 1], and consider that the logical relationship between element *x* and set *S* is no longer simple “either or that”, but use any real value between [0, 1] to represent the “either or that” logical relationship between the element *x* and the set *S*, and the element *x* and the set *T* quantitative fuzzification of the logical relationship between them. Fuzzy mathematics considers that the logical relationship between element *x* and set *S* can be represented by the following fuzzy relationship:

(2) $$\begin{array}{c} S\left(x\right)=\mu \left(x\right)\mu \left(x\right)\in \left[0,1\right],\\ \mu \left(x\right)=\frac{n}{N}, \end{array}$$

where *μ*(*x*) is the degree of membership of the element *x* to the set *S*, and the logical relationship between the element *x* and the set *S* is considered to be a continuous transition process, rather than a sudden jump change, expressed in the form of “membership degree.” The fuzzy relationship between the element x and the set S is greater than the “membership degree.” The more the element x is connected to the set S, the smaller the “membership degree” and the farther the element x is from the set S. That is, the absolute belonging to the relative belonging, using the method of logical operation.

Let a certain factor *Si*.

(3) $$\begin{array}{c} V=\left\{v_{1},v_{2},\ldots ,v_{m}\right\}, \end{array}$$

where *V* is the set of m types of risk states corresponding to the factor *Si*. By determining the membership degree *μj* of each risk state *vj* in the risk state set *V* corresponding to the factor *Si*, the fuzzy judgment matrix of the factor *Si* is constructed.

(4) $$\begin{array}{c} R_{i}=\left\{r_{1},r_{2},\ldots ,r_{m}\right\}, \end{array}$$

where *Ri* is the membership degree *μj* of the factor *Si* corresponding to each risk state *vj* in the risk state set *V*. According to the membership degree *μj* of each risk state *vj* in the risk state set *V* corresponding to a certain factor *S i* in the factor set *S*, the fuzzy judgment matrix *Ri* of the factor *S i* is constructed. Judgment matrix *R i* constructed fuzzy judgment matrix *R* of the factor set. Then, there are

(5) $$\begin{array}{c} R=\left[\begin{array}{c} R_{1}\\ R_{2}\\ .\\ .\\ .\\ R_{n} \end{array}\right], \end{array}$$

where *R* is called the fuzzy judgment matrix of the factor set *S*, and *r ij* represents the degree of membership of the risk state *vj* from the point of view of the factor *S i*. Therefore, the risk state decision set of factor set *S* is

(6) $$\begin{array}{c} B=\left[a_{1},a_{2},\ldots ,a_{n}\right]\left[\begin{array}{c} r_{11}r_{12}\ldots r_{1m}\\ r_{21}r_{22}\ldots r_{2m}\\ \ldots \ldots \ldots \ldots ..\\ r_{n1}r_{n2}\ldots r_{nm} \end{array}\right]. \end{array}$$

In this paper, a combination of triangular distribution and trapezoidal distribution is used to construct the cloud forest tourism safety risk membership function. Then, the security risk membership function of forest tourism is as follows.

Membership functions for class A are as follows:

(7) $$\begin{array}{c} \mu_{A}\left(x\right)=\left\{\begin{array}{c} 0x\leq 70\\ \frac{\left(x-70\right)}{20}70<x\leq 90.\\ 1x\geq 90 \end{array}\right.. \end{array}$$

Membership functions for class B are as follows:

(8) $$\begin{array}{c} \mu_{B}\left(x\right)=\left\{\begin{array}{c} 0x\geq 90\\ \frac{\left(x-90\right)}{20}70<x\leq 90\\ \frac{\left(x-45\right)}{25}45<x\leq 70\\ 0x\leq 45 \end{array}\right.. \end{array}$$

Membership functions for class C are as follows:

(9) $$\begin{array}{c} \mu_{C}\left(x\right)=\left\{\begin{array}{c} 0x\geq 70\\ \frac{\left(70-x\right)}{25}45<x\leq 70\\ \frac{\left(x-15\right)}{30}15<x\leq 45\\ 0x\leq 15 \end{array}\right.. \end{array}$$

Membership functions for class D are as follows:

(10) $$\begin{array}{c} \mu_{D}\left(x\right)=\left\{\begin{array}{c} 0x\geq 45\\ \frac{\left(45-x\right)}{30}\;15<x\leq 45.\\ 1x\leq 15 \end{array}\right.. \end{array}$$

**Figure 3:**
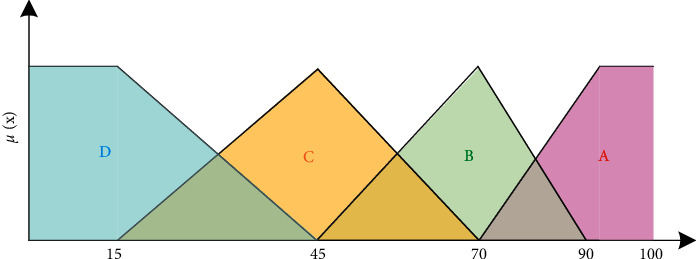
Function of forest tourism safety risk.

Then, the membership function of forest tourism safety risk is shown in Figure 3.

In the form of [*a*, *b*], (*b*, *c*), (*c*, *d*), and [*d*, *e*], the evaluation indicators of different security risk levels are represented by quantitative numerical intervals:

For the evaluation index with a larger value, the smaller the security risk is

(11) $$\begin{array}{c} f=\left\{\begin{array}{c} \frac{20\left(x-d\right)}{e-d}+80\;d\leq x\leq e\\ \frac{20\left(x-c\right)}{d-c}+60\;c\leq x\leq d\\ \frac{30\left(x-b\right)}{c-b}+30\;b\leq x\leq c\\ \frac{30\left(x-a\right)}{b-a}\;a\leq x\leq b \end{array}\right.. \end{array}$$

For the evaluation index with a larger value, the greater the security risk is

(12) $$\begin{array}{c} f=\left\{\begin{array}{c} \frac{20\left(e-x\right)}{e-d}+80\;d\leq x\leq e\\ \frac{20\left(d-x\right)}{d-c}+60\;c\leq x\leq d\\ \frac{30\left(c-x\right)}{c-b}+30\;b\leq x\leq c\\ \frac{30\left(b-x\right)}{b-a}\;a\leq x\leq b \end{array}\right.. \end{array}$$

Up to now, the assessment method is introduced overall, based on which the travel safety design can be discussed in detail.

## 3. Travel Safety Design

**Figure 4:**
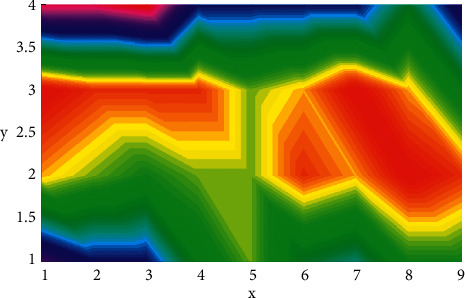
*x* and *y* variation.

Travel safety information refers to information that may endanger the personal or property safety of tourists. When tourists travel to a destination, they are mainly concerned about the scenic spot and its information on geology, meteorology, air quality, water regime, road traffic, public security, epidemic situation, passenger flow, etc. Geological disasters mainly refer to rock loosening, debris flow, etc.; meteorological conditions mainly refer to severe weather such as typhoons, tornadoes, dust storms, thunder and lightning, heavy rain, extremely high and low temperatures such as snow disasters, frost, fierce flames, etc.; air quality mainly refers to those that may affect human health along with air pollution, such as smog, etc.; water conditions mainly refer to sea tides, waves, rivers and mountain streams, flood outbreaks, etc.; road traffic mainly refers to road conditions, traffic congestion, etc.; security conditions mainly refer to whether there are thieves and robbery, including whether there are tour guides slaughtering passengers, forced shopping, etc.; epidemics mainly refer to acute infectious diseases that affect the health of tourists; passenger flow mainly refers to the real-time number of people and passenger flow trends in the entire scenic area and the main browsing points in the scenic area. Such tourism safety information needs to be understood, collected and released by relevant departments in a timely manner and guide tourists to take preventive measures in advance; for tourism management departments, it is an important basis for strengthening tourism safety management and formulating various safety response strategies. The *x* and *y* variation is shown in Figure 4.

Geology, meteorology, air quality, water conditions, road traffic, public security, epidemics, passenger flow, and other information are public safety information, and government departments have the responsibility and obligation to collect and proactively inform the public; tourism administrative departments should establish tourism safety information The information management system is responsible for the collection, analysis, and processing of tourism safety information in various scenic spots under its jurisdiction and timely releases tourism safety warning information, tourism safety rescue information, and tourism safety accident hidden danger information to tourists; tourist attractions should be responsible for the tourism safety of the region. The release of prompt or warning information and the implementation of specific preventive measures are obtained.

The collection of travel safety information serves as the release and early warning of travel safety information. The most important thing in collecting information is the accuracy and timeliness of the data. There are many types of tourism safety information, involving many departments. In addition to the tourism industry itself, it also involves many public business departments, such as land and resources, meteorological and environmental protection, maritime and water affairs, public security and transportation, and forestry and health. Tourism safety information has not yet formed an effective sharing mechanism, and there are still certain obstacles in the channels for tourism authorities to obtain such information, often unable to obtain the required information effectively and timely, and are unable to establish a complete tourism safety information database, which directly affects tourism safety. Therefore, it is necessary to establish an interdepartmental coordination mechanism or a linkage mechanism under the administrative guidance of the government and to establish a sharing platform for the tourism safety information system. The data collected by each scenic spot needs to be obtained by deploying relevant instruments and equipment and applying various acquisition technologies.

**Figure 5:**
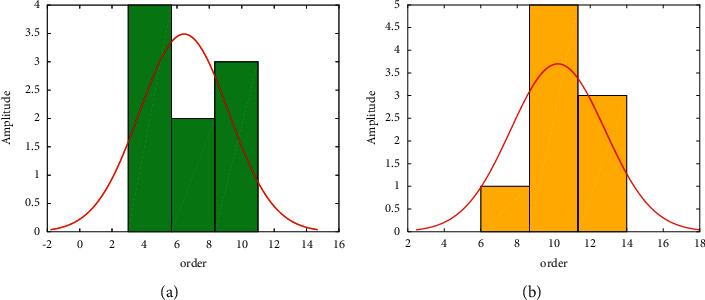
Amplitude. (a) Before optimization. (b) With optimization.

In the design of the scheme, the business functions and system functions of the scenic spots based on the analysis are mainly reflected in the timely acquisition and transmission of information and the support of corresponding functions. The construction requirements can be expressed in three aspects: first is the perception layer, which mainly includes multifunctional information collection terminals, sensors, electronic labels, multimedia information collection, two-dimensional barcodes, real-time positioning, sensor network networking, and collaborative information processing. The problem of information collection is solved. The second is the network layer, which mainly includes the integration of the Internet, mobile communications, and satellite communications to solve the problem of information transmission. The third is the application layer, which mainly includes the construction of the application support platform sublayer and the application service sublayer, especially the application of cloud computing-based intelligent information processing technology and expert decision-making system to solve the application problems of scenic spots. This plan draws on the management model of IoT and the Internet of Things system architecture combined with the tourism security structure. Skynet, ground network, human network, and forest network together form an integrated perception system, which is connected with the “smart forest platform” to form an overall application scheme of “four networks and one platform” for forest tourism, especially for the travel safety of real-time personnel locations. To provide services, Siwang mainly meets the needs of the scenic area's business perspective, and the platform mainly meets the functional level of the scenic tourism security system. The amplitude comparison is plotted in Figure 5. As can be seen, the right one exhibits a better performance since it uses the optimized method.

Besides, it also can be found in Figure 5 that the amplitude is actually different since one is optimized while the other is without optimization. The architecture of the intelligent forestry platform is built, basic platform services such as basic GIS services, data mining services, and data exchange services are developed and built, and a basic environment for interconnection with the four networks and business data integration is built. The business processes and needs of forest safety supervision and tourism services are sorted out, and services including resource supervision, ecological monitoring, tourism resource supervision, personnel management and services, vehicle management, forest fire monitoring, equipment operation and maintenance management, and call center system software are designed and developed. With the help of virtualization technology, the system hardware operating environment is built, and after the system is deployed and debugged, performance and stress tests are carried out to ensure the stable operation of the system.

**Figure 6:**
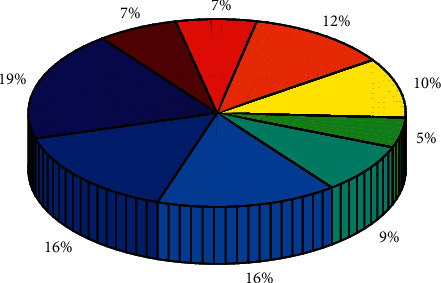
Percentage.

A set of local area networks is built, and the construction content includes the core network system of the computer room and the network system covering other units in the scenic area, to meet the construction of a wired broadband network, especially the network construction of weak nodes such as video surveillance points, protection stations, and forest farms need. The percentage of each part is compared in Figure 6.

The information center computer room provides egress lines to access the Internet through telecommunications. It is necessary to ensure the logical isolation and security protection of the local area network and the Internet and to meet the needs of separation and isolation of internal and external networks.

The perception layer is composed of various perception terminals of the ground network system, the human network system, and the forest network system. The transport layer is composed of a wired broadband network and a mobile communication network, and an ad hoc network is composed of the wireless routing backbone network of the ground network system and the sensor network. Its main function is to realize real-time data transmission between the perception layer and the application layer. The processing layer is composed of middleware such as directory service, management, U-Web service, modeling and management, content management, and spatial information management to support the application layer. The application layer is composed of application systems such as eco-tourism management system, forest resources supervision system, ecological survey monitoring and management system, vehicle management system, comprehensive law enforcement system, comprehensive office system, call center system, video conference system, and operation and maintenance management system. Effective regulation and integrated services for ecotourism and natural resources are clear. The security and comprehensive management system are mainly composed of an information security management system, information security infrastructure, information management and operation and maintenance agencies, etc., to realize the supervision and protection of the information construction, application, operation and maintenance, and safety of scenic spots. The standard specification system is mainly composed of national standards, industry standards, and various technical specifications for the Internet of Things, which provide the scientific basis for the planning, design, construction, management, and operation of the Internet of Things system.

**Figure 7:**
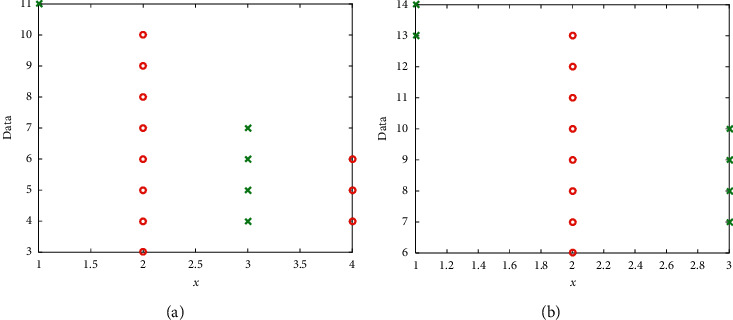
Data variation. (a) Lower data. (b) Higher data.

It mainly builds four networks and one platform. The four networks mainly meet the needs of the business perspective of scenic spots, and the platform mainly meets the needs of the functional level of the tourism security system. Among them, the Skynet system mainly uses the communication and positioning functions of communication satellites, remote sensing satellites, and navigation satellites to obtain remote sensing. The ground network system mainly uses the existing telecommunication broadband and mobile communication network and the sensor network with a two-layer structure is composed of the backbone monitoring network and the local monitoring network to be constructed in this project to realize the detection of scenic spots tourists, vehicles, forest fires, etc. Real-time monitoring and real-time perception are composed of meteorology, hydrology, air quality, surface temperature, and humidity, and timely and accurate data transmission. The human network system mainly uses intelligent positioning badges, mobile multifunctional intelligent handheld terminals, and vehicle-mounted terminals to track the positions of tourists, staff, and tourist vehicles in real time. The forest network system mainly realizes real-time and accurate positioning of tourists, staff, and tourist vehicles through the scientific deployment of forest coordinate equipment. The smart forest platform supports the visual safety supervision and services of forest tourism and natural resources through comprehensive analysis and intelligent data mining of information obtained from SkyNet, GroundNet, HumanNet, ForestNet, and other channels. The data variation is shown in Figure 7. As can be seen, the right subfigure exhibits a better performance since it results in larger data.

## 4. Safety Evaluation

In this section, the safety evaluation is investigated by using the assessment method in Section 2. As is known to all, the scheme evaluation must be closely related to the selection purpose and method. In order to carry out research on the construction method selection of the scheme, deeper research is not only conducted on the scheme itself but also to better guide the better implementation of the scheme, and to serve the implementation. Therefore, it is also the final review of the program research. At present, the methods of decision analysis commonly used at home and abroad mainly include the expert scoring method (i.e., Delphi method), set-valued statistical iteration method, analytic hierarchy process, and fuzzy set-valued statistical method.

**Figure 8:**
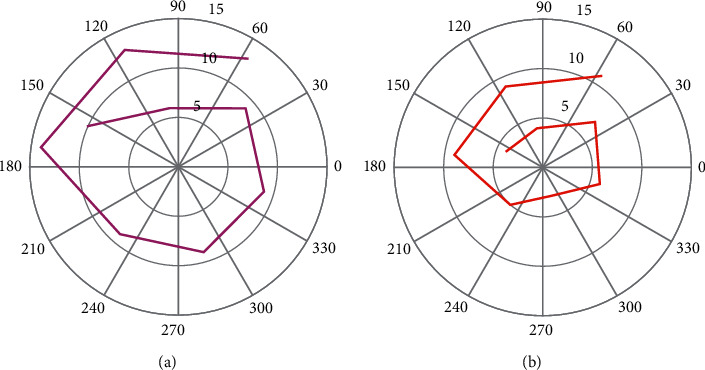
The radar figure. (a) Higher value. (b) Lower value.

On the basis of summarizing the above-mentioned industrial factors for the construction of the scheme, the four criteria of IoT equipment, transmission network, business function, and integration capability have been analyzed above, and the pertinence, applicability, and advanced nature of the construction of the scheme are evaluated. According to the analytic hierarchy process, we can transform this qualitative problem into a quantitative problem to solve. The “9-scale method” proposed by Satie is used to assign values to the comparison results of two different elements, establish a comparative symmetric inverse matrix, and then obtain the weight of each element. In the actual calculation process, the weights between the target layer and the criterion layer, and the criterion layer and the decision layer are calculated, respectively, and then, the connection between the target layer and the decision layer is established to provide the basis for the final decision. It must be emphasized that consistency checks must be performed in applying AHP to ensure the reliability of the results. After analysis and calculation by Yaahp software, the final choice is better than the total integration method for scheme construction. The radar figure is shown in Figure 8.

According to the analysis process of the AHP, the definition of the target layer, the criterion layer, and the program layer is carried out. The model establishment process of this program evaluation is as follows.

### 4.1. Building a Hierarchical Model

The first layer denotes the target layer Z, that is, the evaluation of the plan; the second layer denotes the criterion layer A, that is, IoT device A1, transmission network A2, business function A3, integration capability force A4; the third layer denotes the scheme layer B, that is, the pertinence of the scheme (whether it is suitable for the needs of Jinggangshan Scenic Spot and the on-site environment); B1 denotes applicability of the scheme (whether the scheme has the applicability of promotion and replication); B2 denotes the advancedness of the scheme (the advanced degree of the technology).

The structure is built as shown in Figure 5(b).

What this scheme needs to be evaluated is to consider the four criteria of IoT equipment, transmission network, business function, and integration capability to evaluate the scheme. According to the analytic hierarchy process, this qualitative problem can be transformed into a quantitative problem to be solved. The specific requirements for the evaluation of indicators are as follows.

IoT equipment: the IoT equipment used is advanced and suitable for the needs of scenic spots and the on-site environment.

Transmission network: the advanced nature, stability, and feasibility of the adopted transmission network, as well as the deployment and actual needs of the on-site environment.

Business function: the design of the function according to the needs of the scenic spot meets the needs of the scenic spot, and the function is forward-looking while meeting the needs.

**Figure 9:**
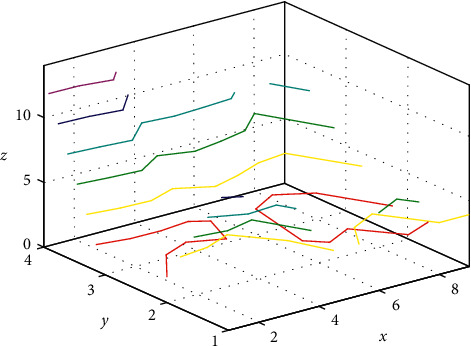
3D contour.

Integration capability: comprehensive implementation integration capability for equipment, resources, and environment is required for solution design, enabling convenient, safe, and efficient deployment. The 3D contour is shown in Figure 9.

## 5. Conclusion

The modern society has paid more attention to the Internet of Things technology, and the Internet of Things is also developing step by step. From the current stage, the application of the Internet of Things can still bring a lot of help in forest management, and to a certain extent, it has promoted the actual management, especially in preventing fires.

## Data Availability

The data used to support the findings of this study are available from the corresponding author upon request.
